# Effects of excitation field amplitude on magnetic particle imaging performance: a modeling study

**DOI:** 10.1088/1361-6463/adeea2

**Published:** 2025-07-22

**Authors:** Ebrahim Azizi, Changzhi Li, Jenifer Gómez-Pastora, Rui He, Kai Wu

**Affiliations:** 1 Department of Electrical and Computer Engineering, Texas Tech University, Lubbock, TX 79409, United States of America; 2 Department of Chemical Engineering, Texas Tech University, Lubbock, TX 79409, United States of America

**Keywords:** magnetic particle imaging, dynamic magnetization, magnetic nanoparticle, excitation field, spatial resolution

## Abstract

Magnetic particle imaging (MPI) is a new tomographic imaging technique that can quantitatively correlate MPI signal intensity to the spatial distribution of magnetic nanoparticle (MNP) tracers. Due to its non-ionizing nature, low background signal from biological matrices, high contrast, and relatively good spatial and temporal resolution, MPI has been actively studied and applied to biomedical imaging and is expected to reach the clinical stage soon. To further improve the spatial resolution limit in MPI, researchers have been working towards optimizing the image reconstruction algorithms, magnetic field profiles, tracer designs, circuitry, etc. Recent studies reported that lower excitation field amplitudes can improve spatial resolution, though this comes at the expense of lower MPI signal and tracer sensitivity. Different excitation field profiles directly affect the collective dynamic magnetizations of tracers recorded by the receiver coil in MPI. However, there is a gap between understanding the relaxation dynamics of MNP tracers, the signal-to-noise ratio (SNR) of MPI signals, and the MPI spatial resolution. In this work, we used a stochastic Langevin equation with coupled Brownian and Néel relaxations to model the magnetic dynamics of different MNP tracers subjected to varying excitation fields. We analyzed the collective time-domain dynamic magnetizations (*M*–*t* curves), magnetic-field hysteresis loops (*M-H* curves), point spread functions (PSFs), higher harmonics, and SNR of the third harmonic to understand how the excitation field affects MPI performance. We employed Full Width at Half Maximum and SNR as evaluation metrics for imaging resolution and signal quality, respectively. Our study supports previous findings on the impact of excitation field amplitude on MPI performance while offering more profound insights into the interplay of nonequilibrium Néel and Brownian relaxation, tracer core size, and SNR.

## 1. Introduction

Magnetic particle imaging (MPI) is a tracer-based, ionizing radiation-free, high contrast, and high sensitivity tomographic imaging technique first reported in 2005 [1]. To date, it has been actively applied to stem cell tracking, cancer imaging, angiography, etc [2–4]. Researchers in this area are actively pursuing the enhancement of the collecting tracer signal and higher spatial-temporal resolution by optimizing several aspects of MPI, such as magnetic nanoparticle (MNP) tracer design, field profile design, image reconstruction method, circuitry design, etc [5–8]. MPI uses a gradient magnetic field, also known as the ‘selection field,’ to selectively saturate the magnetizations of MNP tracers outside a field-free region (FFR). 3D tomographic imaging is achieved by moving the FFR over the subject space. The MPI signal at any given moment is produced by the dynamic magnetic responses of MNP tracers within the FFR and is recorded by the receiver coil. According to the law of induction, the voltage in the receiver coil is linearly proportional to the number of particles in the FFR, making MPI a quantitative tracer imaging technique [9].

Studies have explored the impact of field amplitudes on MPI performance [6, 10–14]. It has been reported that an excitation field with a lower amplitude and a higher frequency can intrinsically improve the spatial resolution of MPI. Although previous studies have addressed the decoupled dependencies of field amplitudes, the collective relaxation effects involving coupled Néel–Brownian dynamics remain unexplored [15, 16]. Additionally, there is a lack of systematic studies on how this field profile affects the dynamic magnetizations of MNP tracers from the viewpoints of energies and torques, and how the collective relaxations and signals of these tracers, together with the intrinsic thermal noise in a dynamical system, influence the MPI performance. A deeper understanding of the interplay of excitation field amplitude and MNP tracers’ dynamic magnetizations, their relaxations, dynamical thermal noise, and MPI signal and resolution will help researchers better customize MNPs for MPI applications.

In this work, we apply a stochastic Langevin equation with coupled Brownian and Néel relaxations to model the dynamic magnetizations of free-standing MNP tracers subjected to an excitation field [17–19]. We assumed an ensemble of 10 000 non-interacting iron oxide MNPs with varying magnetic core sizes was subjected to different excitation fields that have a fixed frequency of 25 kHz and varied amplitude from 5 to 20 mT/*μ*_0_. While Tay *et al* [6] experimentally demonstrated that lower excitation field amplitudes improve resolution and larger core sizes reduce SNR, our simulations, conducted before system-level measurements, reproduce these trends using different evaluation metrics. In contrast to Tay *et al* [14], who emphasized the roles of the relaxation wall and anisotropy in magnetization dynamics, we constrained anisotropy and core size parameters within previously validated parameter ranges to isolate and analyze the specific influence of field amplitude. Compared to the studies by Shasha *et al* [20, 21], which examined how core size affects FWHM across multiple amplitudes, our analysis focuses on how field amplitude affects FWHM across various core sizes, thereby addressing a complementary aspect of the parameters and avoiding redundancy. Moreover, unlike Zhao *et al* [22], whose phenomenological model neglects noise and omits nonequilibrium and coupled relaxation effects, our model incorporates thermal noises (field and torque) and adopts a more realistic dynamic framework. Collectively, our work offers a targeted, noise-inclusive, and complementary perspective on the impact of excitation field amplitude on MPI resolution.

We evaluate our study by collecting time-domain dynamic magnetizations (*M*–*t* curves), magnetization-field hysteresis loops (*M*–*H* curves), point spread functions (PSF curves), and harmonic profiles from these MNP tracers. The FWHM from PSF profiles is used as a parameter to evaluate the intrinsic MPI spatial resolution [10, 23], and the SNR of the 3rd harmonic is used as a parameter to assess the MPI signal.

## 2. Mathematical models

### 2.1. Stochastic Langevin model

The combined Néel and Brownian model tracks the orientations of the magnetization unit vector ${\boldsymbol m}$ and the easy axis unit vector ${\boldsymbol n}$ of an MNP tracer, respectively. The acceleration is neglected since the tracer dynamics are dominated by the viscosity force. The magnetization unit vector ${\boldsymbol m}$ is modeled by the Landau–Lifshitz–Gilbert equation (equation (2)) with a total magnetic field ${\text{H}}$, while the easy axis unit vector ${\boldsymbol n}$ is modeled by defining generalized torque (equation (1)). The general torque and total field are derived from the Helmholtz free energy, ignoring entropic contribution (no tracer agglomeration) and considering the Stoner–Wohlfarth perception for tracers’ internal energy. By defining Néel attempting time ${\tau _0}$ and Brownian relaxation time ${\tau _B}$, the combined model can be written as follows [17, 19, 24]:

\begin{equation*}\frac{{{\text{d}}{\boldsymbol{n}}}}{{{\text{d}}t}} = \left( {\frac{\boldsymbol{\theta} }{{6\eta {V_{\text{h}}}}}} \right) \times {\boldsymbol{n}}\end{equation*}

\begin{equation*}\frac{{{\text{d}}{\boldsymbol{m}}}}{{{\text{d}}t}} = \frac{1}{{{\tau _0}}}\left( {\frac{1}{\alpha }{\boldsymbol{\xi}} + {\boldsymbol{m}} \times {\boldsymbol{\xi}} + \frac{1}{\alpha }{\boldsymbol{{H_{{\text{th}}}}}} + {\boldsymbol{m}} \times {\boldsymbol{{H_{{\text{th}}}}}}} \right) \times {\boldsymbol{m}},\end{equation*}

where ${\boldsymbol \theta}$ is generalized torque, ${\tau _{\text{B}}} = \frac{{3{V_h}\eta }}{{{k_{\text{B}}}T}}$, ${\tau _0} = \frac{{\mu \left( {1 + {\alpha ^2}} \right)}}{{2{k_{\text{B}}}T\alpha \gamma }}$, $\sigma = \frac{{{K_a}{V_c}}}{{{k_{\text{B}}}T}}$ are Brownian relaxation time, Néel attempting time, and the dimensionless ratio of anisotropy energy over thermal energy, respectively. ${V_{\text{h}}}$, $\eta $, ${k_{\text{B}}}$, *T, α, γ*, and ${K_{\text{a}}}$ are the hydrodynamic volume of the tracer, the viscosity of tracer suspension, the Boltzmann constant, temperature, damping parameter, the electron gyromagnetic ratio, and the magnetic anisotropy of the tracer, respectively. $\mu $ is the value of the magnetic moment defined by $\mu = {M_{\text{s}}}{V_{\text{c}}}$ where ${M_{\text{s}}}$ the saturation magnetization of the tracer and ${V_{\text{c}}}$ (=$\frac{\pi }{6}{D_{\text{c}}}^3$) is the magnetic core volume. ${\boldsymbol{H_{{\text{th}}}}}$ and ${\boldsymbol{\theta _{{\text{th}}}}}$ are the thermal noise equivalent field and torque, respectively. The ${\boldsymbol{\xi}} = \frac{{{\mu _0}\mu H}}{{2{k_{\text{B}}}T}} + \sigma \left( {\boldsymbol{{m \cdot n}}} \right){\boldsymbol{n}}$ refers to the dimensionless vector field, and ${\mu _0}$ is the vacuum permeability. The Néel attempting and relaxation times have been extensively explored in [25–29].

The thermal noise correlations can be expressed in a reduced Gaussian form as [17, 19, 24]:

\begin{equation*}\langle {\boldsymbol{\theta _{{\text{th}}}^i}}\left( t \right){\boldsymbol{\theta _{{\text{th}}}^{\,j}}}\left( {t^{\prime}} \right)\rangle = {\tau _{\text{B}}}{\delta _{ij}}\delta \left( {t - t^{\prime}} \right)\end{equation*}

\begin{equation*}\langle {\boldsymbol{H_{{\text{th}}}^i}}\left( t \right){\boldsymbol{H_{{\text{th}}}^{\,j}}}\left( {t^{\prime}} \right)\rangle = {\tau _0}{\delta _{ij}}\delta \left( {t - t^{\prime}} \right),.\end{equation*}

Herein, we consider an ensemble of 10 000 iron oxide MNP tracers and study their magnetization dynamics under varying excitation field amplitudes, mimicking tracers in the FFR of MPI. The numerical solution of the stochastic Langevin equation with the Stranovich interpretation results in the averaged MNP magnetization vector ${\boldsymbol m}$ in the time domain. Since the excitation field is applied along the *z*-direction, we record the magnetizations along the *z*-axis (i.e. ${M_z} = {M_{\text{s}}}{\bar m_z}$), where ${\bar m_z}$ is the *z*-component of the averaged unit magnetization vectors from 10 000 tracers. To this aim, we show the magnetization’s temporal variations along the excitation field direction as *M*–*t* curves and its changes concerning the excitation field as *M*–*H* curves. To compensate for noise, side lobes in frequency, and extensive magnetization ratio at saturation points, we employ signal decimation and Savitzky–Golay smoothing filter with the Kaiser windowing process.

Given the short Néel attempt time (${\tau _0} \approx {\text{ }}$ 9–27 ns), we solved the stochastic differential equation using a high sampling frequency of 750 MHz (corresponding to a time step of approximately 1.34 ns), ensuring that the simulation steps are shorter than the intervals of random Wiener process jumps. However, this fine resolution leads to extremely large values in the magnetization derivative. To manage this, signal decimation was applied by down-sampling to 0.5% of the original sampling rate, followed by a first-order digital low-pass filter with a cutoff frequency set at half of the new sub-sampling rate. While this effectively suppresses high-frequency noise and stabilizes the magnetization derivative, significant noise at sub-sampling frequency ($ \approx 3.75{\text{ MHz}}$) relative to the 25 kHz excitation frequency persists. Moreover, the derivative of the magnetization exhibits sharp peaks (specifically for lower field amplitudes) at saturation points due to edge effects. To address these issues, a Kaiser windowing function (with $\beta = 20$) was applied to both the left and right PSFs, followed by a Savitzky–Golay filter using linear smoothing and a window length of 10. This combination helps further smooth the signal and mitigate remaining high-frequency noise artifacts.

Additionally, following [30], we analyze a 1D MPI system by deriving the induced voltage signal as a convolution process between particle distribution and PSF kernels:

\begin{equation*}u\left( t \right) = - {B_1}\mu \rho \left( z \right)*\dot m\left( {\frac{{{\mu _0}\mu G}}{{2{k_{\text{B}}}T}}z} \right){|_{z = {z_{\text{s}}}}}\frac{{{\mu _0}\mu G}}{{2{k_{\text{B}}}T}}{\dot z_{\text{s}}},\end{equation*}

where $u\left( t \right)$ is the one dimensional signal, $z$ is the spatial dimension where the excitation field is applied, $\rho \left( z \right)$ is the particle density, ${z_{\text{s}}} = \frac{{2{k_{\text{B}}}T}}{{{\mu _0}\mu }}{G^{ - 1}}\xi $, ${{\text{B}}_1}$ is the coil sensitivity (we used 20 mT/A based on experimentally observed values), and $G$ is the strength of the gradient field. As mentioned, the magnetization in equation (5) represents the average normalized magnetization vector obtained from simulating 10 000 particles. We assume this number is sufficient to statistically capture the average behavior of a particle ensemble. When computing the actual signal using equation (5), this magnetization can be rescaled to reflect the real number of particles. After eliminating the fundamental excitation frequency, whose large amplitude compared to higher harmonics can significantly interfere with their accurate extraction, a Fourier transform is applied to isolate the harmonic components. In practical implementations, the suppression of the fundamental harmonic cannot be achieved using a gradiometric coil configuration, which attenuates, but does not completely eliminate, the excitation field component. Alternatively, high-pass or band-pass filtering is often employed in the signal acquisition process to mitigate this issue. However, the detailed design of the signal acquisition system lies beyond the scope of this study.

The resulting harmonics are then analyzed to assess signal quality through SNR evaluation. Furthermore, the MNP tracers are assumed to be ideally concentrated at the field-free point (FFP), modeled as a Dirac delta function. In this scenario, MPI effectively performs an MPS measurement at each FFP position, and spatial imaging is achieved by scanning the FFP across the region of interest. The dynamic magnetization in response to the excitation field along the *z*-axis is plotted as M-H curves over several periods of the excitation field and after stabilization. From these M-H curves, we extract the PSF [30, 31], i.e. the $\dot m\left( {\frac{{{\mu _0}\mu G}}{{2{k_{\text{B}}}T}}z} \right)$ curve). The FWHM of the PSF serves as a metric for assessing the intrinsic spatial resolution in MPI systems, Δ*x*, which can be calculated by [23, 32]:

\begin{equation*}\Delta x = \frac{{{k_{\text{B}}}T}}{{{\mu _0}\mu }}{G^{ - 1}}{\xi _{{\text{FWHM}}}},\end{equation*}

where ${\xi _{{\text{FWHM}}}}$ is the FWHM.

To evaluate the signal quality after digital filtering relative to the underlying model noise, the SNR is calculated by comparing the amplitude of the 3rd harmonic to the thermal torque and field noise levels. The purpose of using FWHM and SNR is to quantitatively assess how Néel and Brownian relaxation, along with thermal field and torque noise, impact MPI signal quality before assessing the MPI system for acquisition. Further investigations into system design and noise effects could build upon this quantification.

### 2.2. Simulation design

In this study, we consider an ensemble of 10 000 free-standing iron oxide MNP tracers in an FFR, exposed to excitation fields of 25 kHz with amplitudes ranging from 5 to 20 mT/*μ*_0_. The tracer solution was assumed to be water or a salt buffer with a viscosity of 1 mPa·s (equivalent to 1 cp) and a temperature of 300 K. We applied the stochastic Langevin equation to study the dynamic magnetizations of MNP tracers with various core sizes. We selected samples with magnetic core sizes of 25 nm, 30 nm, and 35 nm, each coated with a 5 nm thick non-magnetic layer. Due to variations in synthesis methods, oxidation levels, and defects, the saturation magnetizations (Ms) and anisotropy constants (Ka) of iron oxide MNPs can vary widely. The reported Ms for sub-50 nm iron oxide MNPs ranges from 100 to 500 kA/m [33], while Ka values for 10 nm to 30 nm iron oxide MNPs are typically below 20 kJ/m3 [34]. In this work, we collectively assumed the MNP tracers have an Ms of 480 kA/m and a Ka of 7 kJ/m3. To evaluate the impact of field amplitude on MPI resolution, we selected parameter ranges that minimize the influence of other factors, such as ${M_{\text{s}}}$, ${K_{\text{a}}}$, and $\alpha $. Moreover, we represent the particle size as the average size within the MNP ensemble. Although the simulation framework supports the incorporation of particle size distributions, in this study, we adopt a fixed particle size corresponding to the average particle size. The influence of size variability is not explicitly considered here, as it has been addressed in detail in a previous publication [35]. For a more detailed analysis of other parameters, such as ${M_{\text{s}}}$, ${K_{\text{a}}}$, core size, hydrodynamic size, and particle size distribution, readers are referred to our previous studies [35, 36]. All parameters and values used in this work are summarized in table 1.

**Table 1.** Variables and corresponding values are used in this work.

Variable	Values	References
Sampling frequency after signal decimation (MHz)	3.75	—
Excitation field frequency (kHz)	25	—
Viscosity (mPa·s)	1	—
Temperature (K)	300	—
Gradient field (T m^−1^)	1	—
Coil sensitivity (mT A^−1^)	20	—
Damping parameter $\alpha $	1	[18]
Gyromagnetic ratio $\gamma $ (GHz T^−1^)	176	[37]
Magnetic anisotropy K_a_ (kJ/m^3^)	7	[33]
Saturation magnetization M_s_ (kA m^−1^)	480	[33, 38]
Excitation field amplitude (mT/*μ*_0_)	5, 10, 15, 20	[6, 8, 39]
MNP core diameter D_c_ (nm)	25, 30, 35	[40, 41]
MNP Hydrodynamic diameter D_h_ (nm)	35, 40, 45	—

## 3. Results and discussion

### 3.1. Dynamic magnetizations of 25 nm MNP tracers in FFR

**Figure 1.**
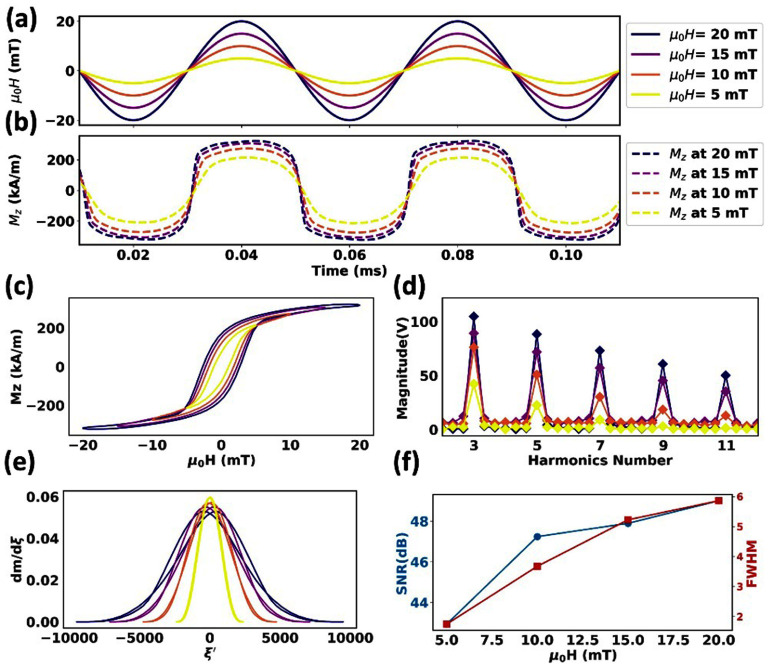
Simulated dynamic magnetization responses of 25 nm MNP tracers subjected to an excitation field of 25 kHz and varying amplitudes from 5 to 20 mT/*μ*_0_. (a) The excitation field is plotted in solid lines, and the corresponding (b) M-t curves are plotted in dashed lines. (c)–(e) are *M*–*H* curves, harmonic profiles, and PSF profiles, respectively. (f) Peak SNR and FWHM profiles are presented as a function of the excitation field amplitude.

Under the assumption that all MNP tracers modeled in this work have identical Ms and Ka, a larger core volume Vc leads to a longer Néel attempting time. Herein, MNP tracers with a 25 nm core size have a shorter Néel attempting time (${\tau _0}$ = 3.37 ns) compared to 30 nm and 35 nm tracers. As a result, the dynamic magnetizations of these 25 nm MNP tracers show a smaller phase delay to the excitation field (figure 1(a) and (b)). At the lowest excitation field amplitude, 5 mT/*μ*_0_, the magnetizations of these 25 nm tracers are not well saturated (cross-comparison of figure 1(b)&(c)), thus, their *M*–*t* curves show semi-sinusoidal shapes. By increasing the excitation field amplitude, the magnetizations of these MNP tracers start to reach their saturation (the MH curves in figure 1(c)), and their *M*–*t* curves become more square-like shapes (figure 1(b)). This behavior results from equation (2) where the Néel attempting time is much shorter than the timing scale of $\xi $, except for the thermal field noise ${H_{{\text{th}}}}$. The short Néel attempting time compared to the Wiener noise causes a polynomial behavior for magnetization. Since ${\tau _0}$ (=3.37 ns) is shorter than the period of the excitation field (=40 $\mu s$), at each instantaneous time, the excitation field is pseudo-constant. Thus, as a result of solving equation (2), the solution, ${\tau _0}^C$, is a polynomial function of time (or of the excitation field), where $C$ is a parameter related to $\xi $. As the excitation field increases, the magnetization continues to follow a polynomial pattern with a larger degree resulting from the excitation field (see dimensionless field in equation (2)). As a result, the *M*–*t* curves become more square-like shapes as the excitation field increases.

The PSF is plotted as ${\text{d}}M/{\text{d}}\xi $ vs. $\xi ^{\prime}$ ($\xi ^{\prime}$ is the first term in $\xi $) curve in figure 1(e). With increasing excitation field amplitude, a larger FWHM is observed (figure 1(f)).

The *M*–*t* curves in figure 1(b) with equation (5) are used to reconstruct the voltage signals from the receiver coil, and harmonics are extracted to plot in figure 1(d). Higher odd harmonics (3 f, 5 f, 7 f, etc.) are uniquely produced by these MNP tracers’ nonlinear responses to the sinusoidal excitation field (frequency *f* = 25 kHz).

The harmonic amplitudes drop with increasing harmonic index. As the excitation field amplitude increases, higher harmonic amplitudes are expected based on equation (5) and the MNPs’ magnetic moments. For evaluating the MPI signals generated by these 10 000, 25 nm MNP tracers, the peak SNR values are summarized in figure 1(f). It is observed that with increasing excitation field amplitude, a higher MPI signal and a worse spatial resolution (based on the FWHM) are expected. Thus, a trade-off between the SNR of the MPI signal and imaging spatial resolution should be made when tuning the excitation field.

### 3.2. Dynamic magnetizations of 30 nm MNP tracers in FFR

**Figure 2.**
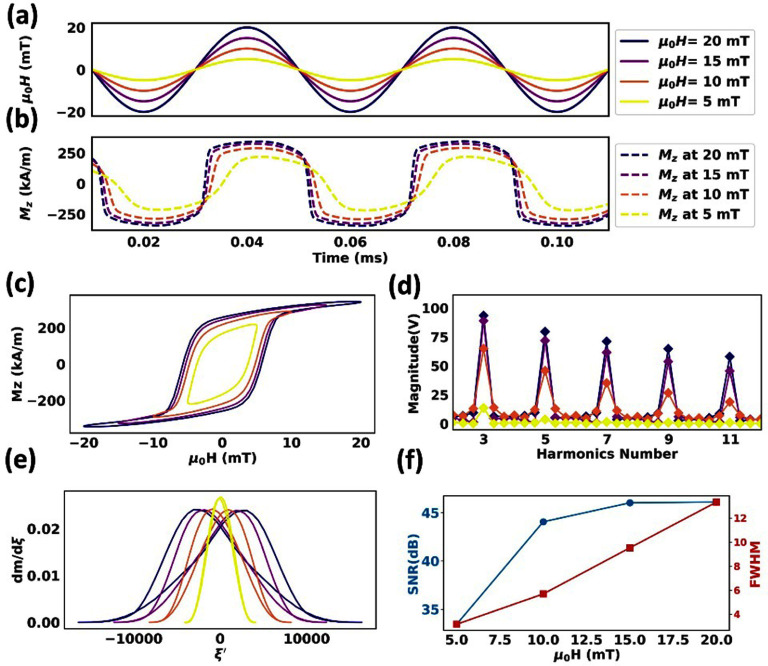
Simulated dynamic magnetization responses of 30 nm MNP tracers subjected to an excitation field of 25 kHz and varying amplitudes from 5 to 20 mT/*μ*_0_. (a) The excitation field is plotted in solid lines, and the corresponding (b) *M*–*t* curves are plotted in dashed lines. (c)–(e) are *M*–*H* curves, harmonic profiles, and PSF profiles, respectively. (f) Peak SNR and FWHM profiles are presented as a function of the excitation field amplitude.

For tracers with 30 nm core sizes, as compared to 25 nm core sizes, the Néel attempting time increases to ${\tau _0}$ =5.82 ns, resulting in larger delayed magnetization dynamics (figure 2(a) and (b)). This delay results in slightly wider hysteresis loops. Additionally, the higher magnetic moment associated with a larger core size slightly enhances the nonlinearity in the *M*–*H* curves (figure 2(c)). At the lowest excitation field amplitude of 5 mT/*μ*_0_, like the 25 nm core-sized tracers, their magnetizations remain near saturation but not yet saturated. The *M*–*t* curve starts to deviate from a sinusoidal shape due to the larger core size and the longer response delay (compare figure 1(b) with figure 2(b) for 5, 10 mT/*μ*_0_ excitation field amplitude). At higher excitation field amplitudes, the tracers reach saturation (the MH curves in figure 2(c)), causing the *M-t* curves to show more square-like shapes. As discussed in section 3.1, this behavior arises from the shorter Néel attempting time relative to $\xi $.

The PSF curves are presented in figure 2(e). As the amplitude of the excitation field increases, a wider PSF is observed. Compared to the 25 nm core-sized tracers, the PSF peaks become less pronounced and the FWHM shows a greater enlargement (compare figure 1(e)&(f) with figure 2(e)&(f)).

Figure 2(d) illustrates the harmonics derived from the receiver coil voltage signals. Like the 25 nm core-sized tracers, higher harmonic amplitudes are also expected with an increase in excitation field for these 30 nm core-sized tracers, according to equation (5). For both 25 nm and 30 nm core-sized MNP tracers, as the excitation field amplitude increases, both the SNR and the FWHM increase. Overall, tracers with a 30 nm core size exhibit a lower SNR compared to those with a 25 nm core, likely due to increased Néel relaxation.

### 3.3. Dynamic magnetizations of 35 nm MNP tracers in FFR

**Figure 3.**
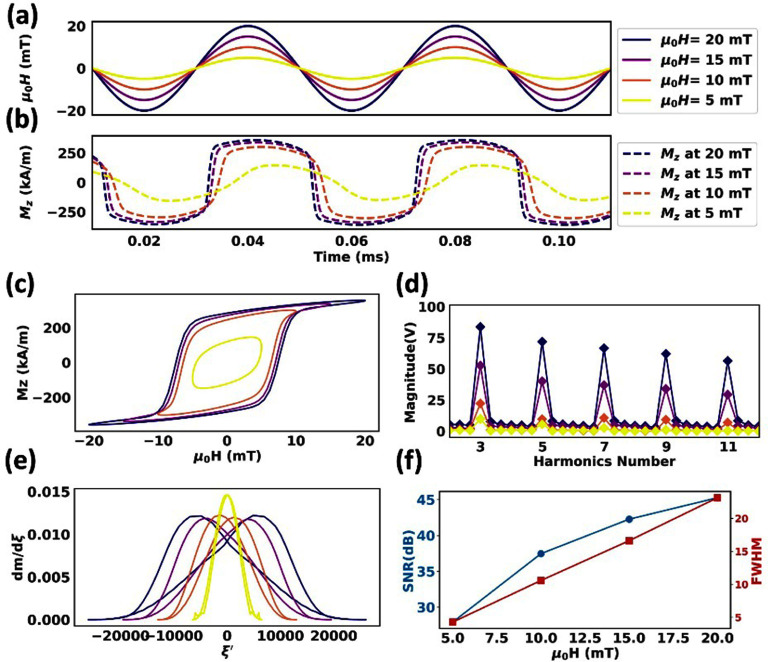
Simulated dynamic magnetization responses of 35 nm MNP tracers subjected to an excitation field of 25 kHz and varying amplitudes from 5 to 20 mT/*μ*_0_. (a) The excitation field is plotted in solid lines, and the corresponding (b) *M*–*t* curves are plotted in dashed lines. (c)–(e) are *M*–*H* curves, harmonic profiles, and PSF profiles, respectively. (f) Peak SNR harmonic and FWHM profiles presented as a function of the excitation field amplitude.

The larger core size exhibits similar effects on the dynamic magnetizations, harmonic signals, and PSF profiles of tracers for 35 nm core-sized MNPs, as described in the previous sections. For these 35 nm core-sized tracers, an increased Néel attempting time of ${\tau _0}$ =9.24 ns results in a greater delay in the *M*–*t* curves (figure 3(a) and (b)) and wider hysteresis in the MH curves along with higher nonlinearity (figure 3(c)). As the excitation field amplitude increases, the magnetizations of tracers reach saturation (figure 3(c)), driving the *M*–*t* curves toward more square-like shapes (figure 3(b)).

Like the trend observed in 25 nm and 30 nm core size tracers, the PSF peaks shrink for 35 nm tracers under higher excitation field amplitudes, which increment FWHM (figure 3(e)). The PSFs are wider when compared to smaller core sizes (25 and 30 nm). Additionally, harmonics gradually decrease with a higher harmonic index (figure 3(d)) and are weaker compared to 25 and 30 nm core sizes. The FWHM and SNR trends are like those observed for core sizes of 25 and 30 nm, but with lower SNR values and higher FWHM in comparison (figure 3(f)).

Table 2 summarizes the core sizes, FWHM, and SNR at the third harmonic for the three types of particles investigated in this study. The variations in FWHM and SNR are illustrated for each core size, and the average values are considered to highlight the differences among the core sizes.

**Table 2.** Summary of tracer core sizes, FWHM, and SNR at the 3rd harmonic, as a function of excitation field amplitude.

Dc (nm)	Field amplitude (mT/*μ*_0_)	FWHM	SNR (dB)	Average SNR (dB)	Average FWHM^a^
25	5	1.74	42.94	46.77	4.12
	10	3.67	47.24		
	15	5.22	47.89		
	20	5.86	49.00		

30	5	3.17	33.45	42.41	7.93
	10	5.69	44.06		
	15	9.52	46.02		
	20	13.32	46.09		

35	5	4.27	27.92	38.23	13.65
	10	10.58	37.46		
	15	16.61	42.27		
	20	23.12	45.26		

^a^ FWHM is dimensionless, the dimensionality of FWHM can be achieved by equation (5).

## 4. Conclusions

The excitation field is a crucial element in the design of MPI systems, yet it has garnered limited attention from researchers until recently. Although the influence of the excitation field has been previously examined and experimentally validated within the limited range where linear models, such as the one used by [42], are applicable, these models rely on simplifying assumptions like negligible Néel relaxation and a linear response regime, often maintained through the use of a bias field. However, comprehensive studies under more general alternating field conditions, especially those incorporating both Néel and Brownian relaxation mechanisms and accounting for anisotropy energy, remain incomplete. Such advanced models provide a more accurate description of magnetization dynamics, particularly when the field amplitudes or particle properties fall outside the linear regime assumed by earlier phenomenological approaches. In this study, a stochastic Langevin equation was employed to model an ensemble of 10 000 MNP tracers with varying magnetic core sizes under different excitation field amplitudes. The resulting dynamic magnetizations were plotted as a function of time and the excitation field. Furthermore, the PSF profiles and higher harmonics were examined. The FWHM was derived from the PSF and used as an indicator of spatial resolution in MPI [23, 32]. Harmonics, derived from the voltage signals according to Faraday’s law of induction, were used as a metric for MPI signal quality. The SNR at the 3rd harmonic and FWHM from the PSF were assessed at various excitation field amplitudes.

Our findings reveal that for tracers with smaller core sizes, their shorter Néel attempting time minimizes the magnetization delay to the excitation field. As a result, the *M*–*t* curves transition from semi-sinusoidal to more square-like shapes as the excitation field amplitude increases. As the core size increases, the Néel attempting time increases, leading to a greater phase delay in magnetization dynamics and a wider hysteresis in the *M*–*H* curves. While tracers with larger core sizes produce stronger magnetic moments in response to the excitation field, they also experience greater phase delays due to longer relaxation times. These competing effects shape both the hysteresis behavior and the PSF profiles. These results indicate that the relaxation time of tracers has a more dominant impact on FWHM and SNR than core size or the tracer’s response to the field. Increasing the excitation field amplitude does not overcome the effects of relaxation; instead, it further degrades imaging resolution. Therefore, optimal imaging resolution can be achieved with a lower excitation field amplitude and using tracers with smaller core sizes. However, this trade-off differs for signal quality. SNR decreases with larger core sizes but increases with excitation field amplitude. The SNR improvement for a specific core size comes at the expense of spatial resolution, as both larger core sizes and higher excitation field amplitudes widen the FWHM.

In summary, for all the tracers modeled in this work, larger FWHM values were observed with higher excitation field amplitudes, indicating that MPI spatial resolution worsens as excitation field amplitude increases, which is consistent with the reported literature [6, 10, 11, 13]. Our results align with the findings reported in [6, 14, 20, 21, 35], confirming that lower excitation field amplitudes enhance resolution and larger core sizes reduce SNR. In addition, our simulations provide more detailed insight into the impact of field amplitude by isolating it from other factors and incorporating thermal noise and coupled relaxation dynamics. These findings highlight the crucial interplay between excitation field strength and tracer core size in determining MPI imaging resolution. In summary, achieving an optimal balance between SNR, spatial resolution, and tracer core size is essential, as smaller cores enhance imaging quality but require precise adjustment of the excitation field to optimize MPI performance. If the SNR has large enough values, then a lower excitation field and smaller core size favor higher MPI resolution. Once the SNR becomes crucial, the adjustment of the SNR and excitation fields needs to be considered.

## Data Availability

All data that support the findings of this study are included within the article (and any supplementary files).
